# Mating Systems of the Tree-Killing Bark Beetle *Polygraphus proximus* (Coleoptera: Curculionidae: Scolytinae)

**DOI:** 10.1093/jisesa/ieaa140

**Published:** 2020-12-25

**Authors:** Kenta Köbayashi, Etsuro Takagi

**Affiliations:** Department of Tourism Science, Graduate School of Urban Environmental Sciences, Tokyo Metropolitan University, Minami-Osawa, Hachioji, Tokyo, Japan

**Keywords:** *Abies*, gallery arms, monogyny, re-emergence

## Abstract

*Polygraphus proximus* Blandford (Coleoptera: Curculionidae: Scolytinae) has caused mass mortality of fir (*Abies* spp. (Pinaceae)) forests across large areas of Russia in the past decade. More recently, mass mortality of *A*. *veitchii*  Lindl. due to *P*. *proximus* infestation has been reported in Japan. This bark beetle species traditionally has been considered to be polygynous because their galleries have multiple gallery arms, and because harem-polygyny is common in the tribe Polygraphini. Although the mating system(s) potentially could have a marked effect on their reproductive success and population dynamics, the reproductive behavior of the tree-killing bark beetle *P*. *proximus* has not been investigated in detail in a natural setting in Japan. We, therefore, investigated the number of males and females in a gallery and the number of gallery arms in *Abies* species in Japan. None of the galleries examined contained more than one male, and 57.2% of the galleries had multiple gallery arms, even though only 2.8% of the galleries contained two females. The findings showed that the typical mating system employed by *P*. *proximus* is monogyny and that this species constructs multiple gallery arms in each gallery. In addition, 70.4% of galleries in which the sex of adult beetles could be determined contained no males, and 26.6% contained no females, suggesting that *P*. *proximus* males and females re-emerge.

Bark beetles (Coleoptera: Curculionidae: Scolytinae) cause a great deal of damage to coniferous forests around the globe (Lausch et al. 2013, USDA Forest Service 2017). In addition to causing widespread tree mortality, they can change the structure, composition, and function of forests when they become numerous (Allen et al. 2006, Raffa et al. 2008, Bentz et al. 2015, Morris et al. 2018).

*Polygraphus proximus* Blandford (Coleoptera: Curculionidae: Scolytinae) has become a striking example of biological invasion in Russia (Kerchev 2014a, Kharuk et al. 2019). Specifically, the beetle has invaded European Russia and West Siberia, where it has caused mass mortality across extensive areas of both planted and natural fir forests in the last decade (Baranchikov et al. 2011, Kononov et al. 2016). In Japan, *P*. *proximus* has caused extensive damage to *A*. *firma* Sieb. et Zucc. (Pinaceae) trees that have been weakened by the curculionid, *Parendaeus abietinus* Kojima and Morimoto (Coleoptera: Curculionidae) (Tokuda et al. 2008). More recently, the mass mortality of artificially planted *A*. *veitchii* Lindl. trees by *P*. *proximus* has been reported in Japan (Takagi et al. 2018, 2021).

The mating systems employed by bark beetles show remarkable diversity, and can be classified by the number of females that breed simultaneously with the same male (Kirkendall 1983, Kirkendall et al. 2015). These mating systems may influence the reproductive success and population dynamics of the bark beetles (Kirkendall 1983). For instance, an increase in the number of females in a gallery may often result in a decrease in larval survivorship due to resource competition (Beaver 1974, Kirkendall 1989, Sallé and Raffa 2007). An increase in the number of females in a gallery may also cause a reduction in the number of eggs laid per female (Latty et al. 2009). Males can copulate with multiple females (Robertson 1998). In addition, when only one female excavates a gallery, gallery arms can be used as a proxy for the reproductive capacity and characteristics of the female, such as number of eggs laid. However, a lack of information on mating systems in these beetles makes it difficult to estimate the reproductive potential and the fecundity of females (Kirkendall 1983).

The dominant mating system among members of the tribe Polygraphini is harem-polygyny (Kirkendall 1983, Ueda et al. 2009). Since *P*. *proximus* typically constructs galleries with two or more gallery arms (Kabe 1959, Nobuchi 1980, Tokuda et al. 2008, Krivets 2012), it has been suggested that they have a harem-polygyny mating system (Kabe 1959, Yamaguchi 1963, Nobuchi 1980, Krivets 2012). However, a recent laboratory experiment showed that *P*. *proximus* was monogynous in *A*. *sibirica* Ledeb. (Kerchev 2014b), but no detailed information was collected on whether the same mating system was employed in a natural setting. In that study, only one *P*. *proximus* female, which was housed in a small plastic tube with one male and one or more females, colonized *A*. *sibirica* logs with the male and excavated gallery arms (Kerchev 2014b). However, such high densities rarely occur in natural settings, and it has been shown that the mating systems employed in natural settings can differ markedly from laboratory observations (Reid 1999). In addition, the mating systems employed by *P*. *proximus* in *Abies* spp. in Japan have not been investigated, even though mass mortality of fir trees has been observed recently in Japan (Takagi et al. 2018, 2021). Increased understanding of these mating systems may lead to more efficient control methods, such as predicting population trends and interfering with beetle reproduction, being devised and implemented. To demonstrate the mating systems of *P*. *proximus* in all five *Abies* spp. in Japan, we investigated the number of males and females in a gallery, as well as the number of gallery arms excavated by this beetle.

## Materials and Methods

### Study Site

Two study sites were selected in central subalpine/subarctic forests on Honshu and Hokkaido, Japan. One was located in the Botanical Gardens of the Yatsugatake-Kawakami Forest Station (35° 94′ N, 138° 47′ E), Mountain Science Center, University of Tsukuba, Nobeyama Highland, Minamisaku County, Nagano Prefecture, Japan (YKF). The other was located in the Botanical Gardens of The University of Tokyo Hokkaido Forest (43° 15′ N, 142° 30′ E), The University of Tokyo, Furano City, Hokkaido, Japan (UTHF).

### Bark Beetles

*Polygraphus proximus* is a nonaggressive phloephagous bark beetle that feeds on the following Far Eastern fir species: *Abies firma*, *A*. *holophylla* Maxim., *A*. *homolepis* Sieb. & Zucc., *A*. *mariesii* Masters, *A*. *nephrolepis* (Trautv. ex Maxim.) Maxim., *A*. *sachalinensis* (Fr. Schmidt) Masters, *A*. *sibirica*, and *A*. *veitchii* (Nobuchi 1966, Koizumi 1977, Kerchev 2014a). The beetles are native to northeastern China, Korea, Japan, and the southern part of the Russian Far East (Nobuchi 1966, Kerchev 2014a).

### Fir Trees

*Abies* species are coniferous evergreen trees that grow to a height of 25–30 m. Five *Abies* species are native to Japan and typically grow allopatrically in Japan. *Abies sachalinensis* is the only *Abies* species distributed in Hokkaido Island, Japan, while *A*. *veitchii*, *A*. *mariesii*, *A*. *firma*, and *A*. *homolepis* are not in Hokkaido.

### Field Survey

To determine the number of males and females in a gallery and the number of gallery arms in a gallery, we examined infested logs at both study sites. For each host species, four to eight noninfested standing trees (diameter at breast height [DBH]: 14–20 cm) were felled. *Abies sachalinensis* trees were felled at UTHF on 15 April 2019. *Abies homolepis* trees were felled at Sugadaira Research Station, the Mountain Science Center, University of Tsukuba, Ueda City, Nagano Prefecture, Japan on 17 April 2019. *Abies veitchii* trees were felled at YKF on 18 April 2019. *Abies firma* trees were felled in The University of Tokyo Chiba Forest, The University of Tokyo, Kamogawa City, Chiba Prefecture, Japan on 24 April 2019. *Abies mariesii* trees were felled in the Education and Research Center of Alpine Field Science, the Shinshu University, Ina County, Nagano Prefecture, Japan on 13 May 2019. The trees were cut into logs measuring 1 m in length. To prevent drying, paraffin was applied to both cut ends of the logs. Three logs of each species (diameter: 11–18 cm) were then randomly selected for each experiment. Three logs of *A*. *sachalinensis*, which is the only native *Abies* species to Hokkaido Island, were placed at UTHF on 22 May 2019, and three logs of each of the four remaining fir species (i.e., 12 logs), which are not native to Hokkaido Island but to Honshu, were placed at YKF on 16 May 2019. The remaining logs were used for other studies.

The bark from the logs at UTHF was then peeled on 16 and 17 July 2019, and at YKF on 8 July 2019. The beetles in each gallery were collected and placed individually in a plastic tube for sexual identification, which was based on secondary sexual characters and performed under a stereomicroscope (Kerchev 2014b). Photographs were taken of the bark that was removed in the field, and the number of gallery arms in each gallery were counted by referring to photographs. Fisher exact test was used to compare the proportion of galleries with no males or females using the software package R 3.3.2 (R core team 2016).

## Results

In total, 342 galleries were identified in the 15 logs (Tables 1 and 2). Although all galleries contained eggs and 323 (94.4%) galleries contained larvae, no pupae or new adults were found in the logs. None of the intact galleries contained more than one male; the sex of the beetles could not be determined in 45 galleries as these were damaged during bark removal (Table 1). Of the 218 galleries containing females, 212 (97.2%) contained one female and six (2.8%) contained two females (Table 1). None of the galleries contained more than two females. Of the 48 galleries that contained both males and females, 47 contained one male and one female and only one gallery contained two females with one male (Table 1).

**Table 1.** Number of galleries containing male and female *P*. *proximus* in *A*. *mariesii*, *A*. *veitchii*, *A*. *homolepis*, *A*. *firma*, and *A*. *sachalinensis*

Study site	Tree species	Log code	No. of galleries							
			One male	One female	One male and one female	Two females	One male and two females	No adults	Unclear	Total
YKF	*A*. *mariesii*	O1-4	4	13	8	0	0	0	1	26
		O2-3	1	6	4	1	1	0	2	15
		O3-2	5	23	10	1	0	7	4	50
	*A*. *veitchii*	S2-1	0	0	0	0	0	0	0	0
		S4-1	0	2	0	0	0	0	2	4
		S4-6	0	1	0	0	0	0	0	1
	*A*. *homolepis*	U2-2	0	0	0	0	0	0	0	0
		U4-6	0	0	0	0	0	0	0	0
		U6-5	0	1	0	0	0	0	0	1
	*A*. *firma*	M1-1	0	0	0	0	0	0	0	0
		M2-4	0	2	1	0	0	0	1	4
		M4-1	0	0	0	0	0	0	0	0
UTHF	*A*. *sachalinensis*	T2-4	8	46	17	2	0	14	8	95
		T5-1	13	40	5	0	0	13	17	88
		T6-5	9	31	2	1	0	5	10	58
Total			40	165	47	5	1	39	45	342

**Table 2.** Number of galleries having different number of gallery arms in each gallery of *P*. *proximus* in *A*. *mariesii*, *A*. *veitchii*, *A*. *homolepis*, *A*. *firma*, and *A*. *sachalinensis*

Study site	Tree species	Log code	No. of galleries				
			One	Two	Three	Unclear	Total
YKF	*A*. *mariesii*	O1-4	10	16	0	0	26
		O2-3	7	8	0	0	15
		O3-2	9	40	1	0	50
	*A*. *veitchii*	S2-1	0	0	0	0	0
		S4-1	4	0	0	0	4
		S4-6	1	0	0	0	1
	*A*. *homolepis*	U2-2	0	0	0	0	0
		U4-6	0	0	0	0	0
		U6-5	1	0	0	0	1
	*A*. *firma*	M1-1	0	0	0	0	0
		M2-4	4	0	0	0	4
		M4-1	0	0	0	0	0
UTHF	*A*. *sachalinensis*	T2-4	47	39	2	7	95
		T5-1	34	46	3	5	88
		T6-5	20	27	1	10	58
Total			137	176	7	22	342

In total, 183 (57.2%) galleries had more than one gallery arm; the number of arms could not be determined for 22 galleries, which were damaged during bark removal (Table 2).

The proportion of galleries where males were absent (209/297) was significantly higher than that of the galleries where females were absent (79/297) (Fisher exact test; *P* < 0.001).

## Discussion

The present study examined the number of male and female *P*. *proximus* beetles in galleries in the field. None of the galleries contained more than one male. Only 2.8% of the galleries contained two females, while 57.2% of the galleries had multiple gallery arms.

Previous studies have proposed that *P*. *proximus* employed harem-polygyny based on the presence of multiple gallery arms (Kabe 1959, Yamaguchi 1963, Nobuchi 1980, Krivets 2012). Our findings suggest that *P*. *proximus* is essentially monogynous and excavates several gallery arms in each gallery in *A*. *sachalinensis* and *A*. *mariesii* native to Japan. This conclusion is supported by laboratory experiments using *A*. *sibirica* in which *P*. *proximus* produced multiple gallery arms despite only one female being observed in each gallery (Kerchev 2014b). Since *A*. *veitchii*, *A*. *homolepis*, and *A*. *firma* were rarely attacked, further studies on the three species should be conducted.

A previous study reported that the mean number of offspring *P*. *proximus* adults per gallery of windfallen *A*. *sachalinensis* was 46.0, and estimated 23.0 per female because two females were considered to construct a gallery (Yamaguchi 1963). However, our results showed that multiple gallery arms in a gallery were constructed by only one female in most cases. Thus, our results indicate the fecundity of *P*. *proximus* is much higher than previously thought.

The most widespread mating system among members of the tribe Polygraphini is harem-polygyny (Kirkendall 1983, Ueda et al. 2009). In polygynous bark beetles, males commonly initiate construction of the galleries (Kirkendall 1983). Conversely, in monogynous bark beetles, which is the ancestral mating system, it is the females that typically initiate gallery construction (Kirkendall 1983), and the males stridulate to deter other males from entering the galleries initiated by females (Liu et al. 2020). *Polygraphus rufipennis* Kirby has a polygynous mating system and their males show a greater tendency to initiate gallery construction compared to females, but the males stridulate (Rudinsky et al. 1978). On the other hand, the present study showed that *P*. *proximus* was essentially monogynous, and only a few galleries contained two females. Either sex of *P*. *proximus* may initiate galleries, and the males stridulate (Kerchev 2019). These may suggest that *Polygraphus* spp. is an evolutionary holdover from the transition from monogyny to harem-polygyny.

A previous study showed that the galleries of *P*. *proximus* remained occupied by both sexes until the end of the egg-laying process (Kerchev 2014b). However, the present study showed the galleries without males (70.4%) and/or females (26.6%), although multiple galleries contained eggs and/or larvae. Additionally, the galleries contained significantly more females than males. These results suggest that *P*. *proximus* re-emerges, and the males leave the galleries before the females.

In many other bark beetle species, the males and females can re-emerge to reproduce in other hosts in the same breeding season (Kirkendall 1983). Conversely, the males that remain with their mated females in the galleries can increase reproductive success by parental cares, such as removing the frass and protecting the brood from predators (Robertson 1998, Robertson and Roitberg 1998, Baruch et al. 2017). To improve the understanding of *P*. *proximus* population dynamics, further studies on the confirmation of re-emergence and construction of more than one gallery system, as well as the reproductive behavior of *P*. *proximus* in the gallery, should be conducted.
